# Thermal decomposition of the amino acids glycine, cysteine, aspartic acid, asparagine, glutamic acid, glutamine, arginine and histidine

**DOI:** 10.1186/s13628-018-0042-4

**Published:** 2018-02-09

**Authors:** Ingrid M. Weiss, Christina Muth, Robert Drumm, Helmut O. K. Kirchner

**Affiliations:** 1 Institute of Biomaterials and Biomolecular Systems, University of Stuttgart, Pfaffenwaldring 57, Stuttgart, D-70569 Germany; 2 INM-Leibniz Institute for New Materials, Campus D2 2, Saarbruecken, D-66123 Germany

**Keywords:** Amino acid, Thermal analysis, Quantitative mass spectrometry

## Abstract

**Background:**

The pathways of thermal instability of amino acids have been unknown. New mass spectrometric data allow unequivocal quantitative identification of the decomposition products.

**Results:**

Calorimetry, thermogravimetry and mass spectrometry were used to follow the thermal decomposition of the eight amino acids G, C, D, N, E, Q, R and H between 185 °C and 280 °C. Endothermic heats of decomposition between 72 and 151 kJ/mol are needed to form 12 to 70% volatile products. This process is neither melting nor sublimation. With exception of cysteine they emit mainly H_2_O, some NH_3_ and no CO_2_. Cysteine produces CO_2_ and little else. The reactions are described by polynomials, AA→*a* NH_3_+*b* H_2_O+*c* CO_2_+*d* H_2_S+*e* residue, with integer or half integer coefficients. The solid monomolecular residues are rich in peptide bonds.

**Conclusions:**

Eight of the 20 standard amino acids decompose at well-defined, characteristic temperatures, in contrast to commonly accepted knowledge. Products of decomposition are simple. The novel quantitative results emphasize the impact of water and cyclic condensates with peptide bonds and put constraints on hypotheses of the origin, state and stability of amino acids in the range between 200 °C and 300 °C.

## Background

The so-called 20 standard amino acids are fundamental building blocks of living systems [1]. They are usually obtained in solid form from aqueous solution by evaporation of the solvent [2]. Most of today’s knowledge about amino acids is therefore limited to the temperature and pressure range of liquid water. A huge number of physical and chemical data were unequivocally established − at least for the 20 standard amino acids [3]. Thermal stability or instability of amino acids, however, is one of the few fields which remains speculative until today, at least to some extent. One major reason for deficiencies in that respect could be that data aquisition is usually performed without analyzing the entire system, where liquid amino acids and decomposition products, as well as their respective gas phases must be taken into account. We used a commercial thermal analysis system with a direct transfer line to a mass spectrometer for characterizing the melting or decomposition process of amino acids under inert atmosphere in the temperature range between 323−593 K and detection of masses between 1−199 Da in the vapour phase. Mass analysis was calibrated with respect to NH_3_, H_2_O and CO_2_ by searching for suitable reference substances, with the goal to identify whether or not there is a common underlying principle of melting − solidification and/or sublimation − desublimation and/or irreversible decomposition for amino acids. Previous reports missed the quantitative identification of gaseous products. The broader implication of this general relationship between amino acids and their condensation products is that amino acids might have been synthesized under prebiological conditions on earth or deposited on earth from interstellar space, where they have been found [4]. Robustness of amino acids against extreme conditions is required for early occurrence, but little is known about their nonbiological thermal destruction. There is hope that one might learn something about the molecules needed in synthesis from the products found in decomposition. Our experimental approach is not biochemical, it is merely thermochemical.

## Methods

### DSC, TGA, QMS

Altogether 200 samples of amino acids of at least 99.99% purity from Sigma-Aldrich were tested in a Simultaneous Thermal Analysis apparatus STA 449 Jupiter (Netzsch, Selb, Germany) coupled with Mass spectrometer QMS 403C Aëolos (Netzsch). Specimens of typically 10 mg weight in Al cans were evacuated and then heated at 5 K/min in argon flow. Differential scanning calorimetry (DSC) and thermal gravimetric analysis/thermogravimetry (TGA, TG), as well as quantitative mass spectrometry (QMS) outputs were smoothed to obtain the data of “Raw data” section. The mass spectrometer scanned 290 times between 30 °C and 320 °C, i.e. at every single degree in 1 Da steps between 1 Da and 100 Da. Alltogether, 290×100×200=5.8 million data points were analyzed.

### Visuals

A MPA120 EZ-Melt Automated Melting Point Apparatus (Stanford Research Systems, Sunnyvale, CA, U.S.A.) equipped with a CAMCORDER GZ-EX210 (JVC, Bad Vilbel, Germany) was used for the optical observations. The same heating rate of 5 K/min was employed, but without inert gas protection. Screen shot images were extracted from continuous videos registered from 160 to 320 °C, for all amino acids significant moments are shown in “Results”.

## Results

### Raw data

**Fig. 1:**
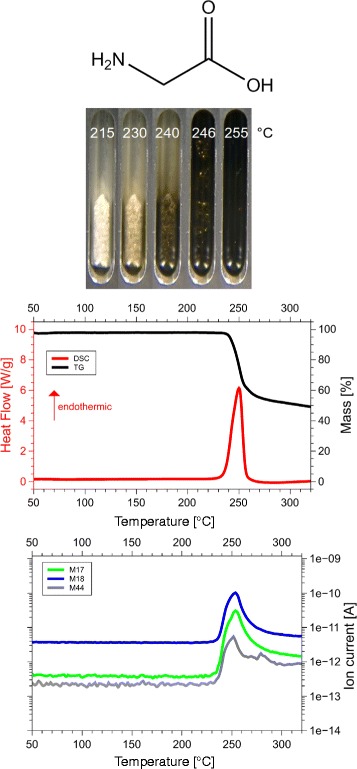
Glycine data. *C*_2_*H*_5_N*O*_2_, 75 Da, *H*_*f*_=−528 kJ/mol

**Fig. 2:**
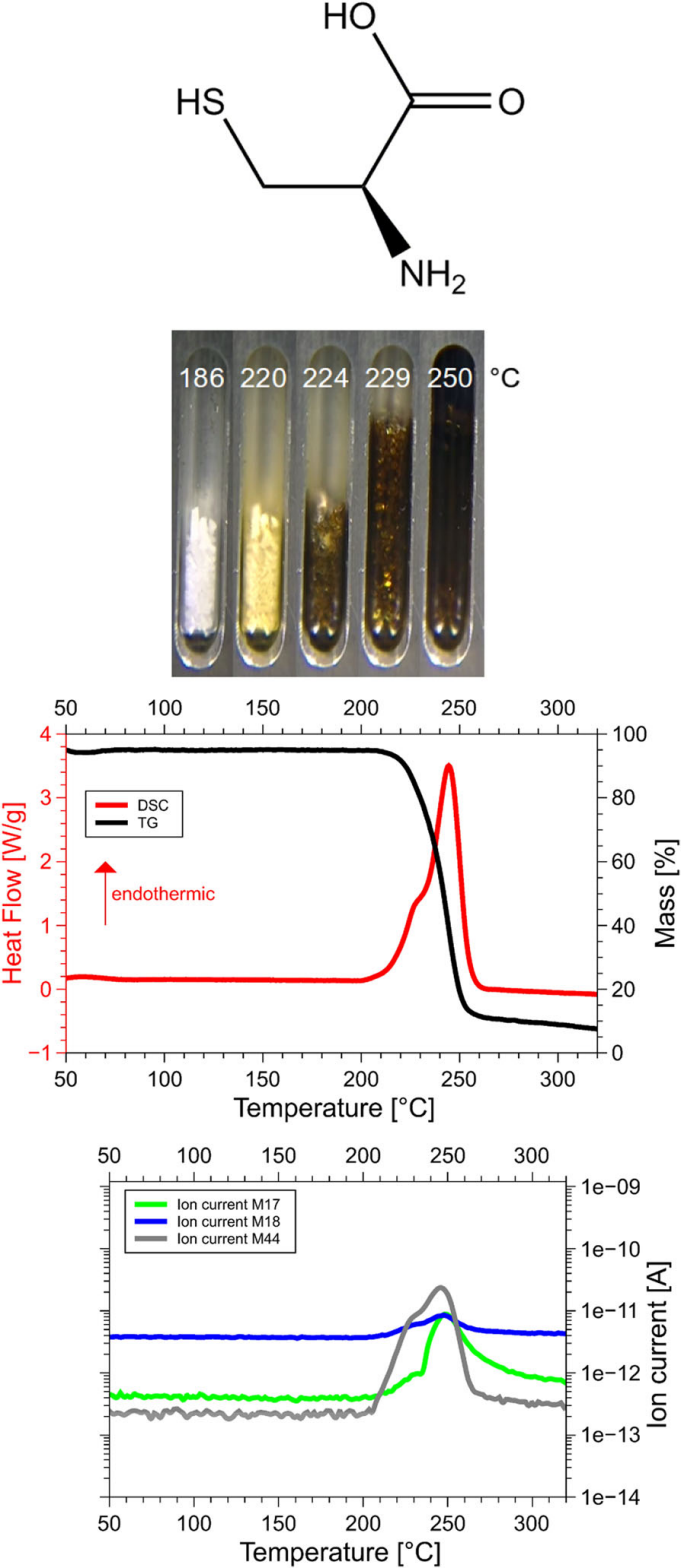
Cysteine data. *C*_3_*H*_7_N*O*_2_S, 121 Da, *H*_*f*_=−534 kJ/mol

**Fig. 3:**
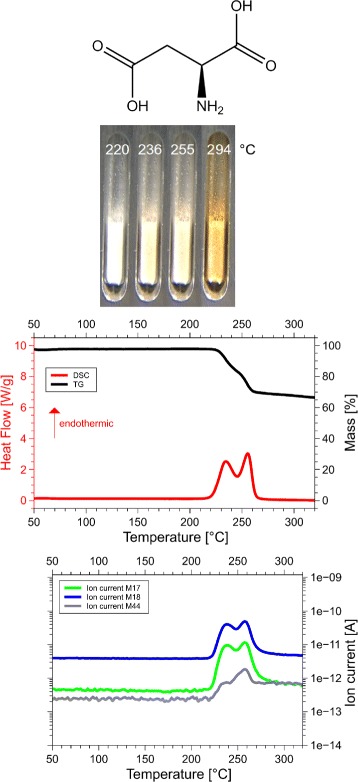
Aspartate data. *C*_4_*H*_7_N*O*_4_, 133 Da, *H*_*f*_=−973 kJ/ mol

**Fig. 4:**
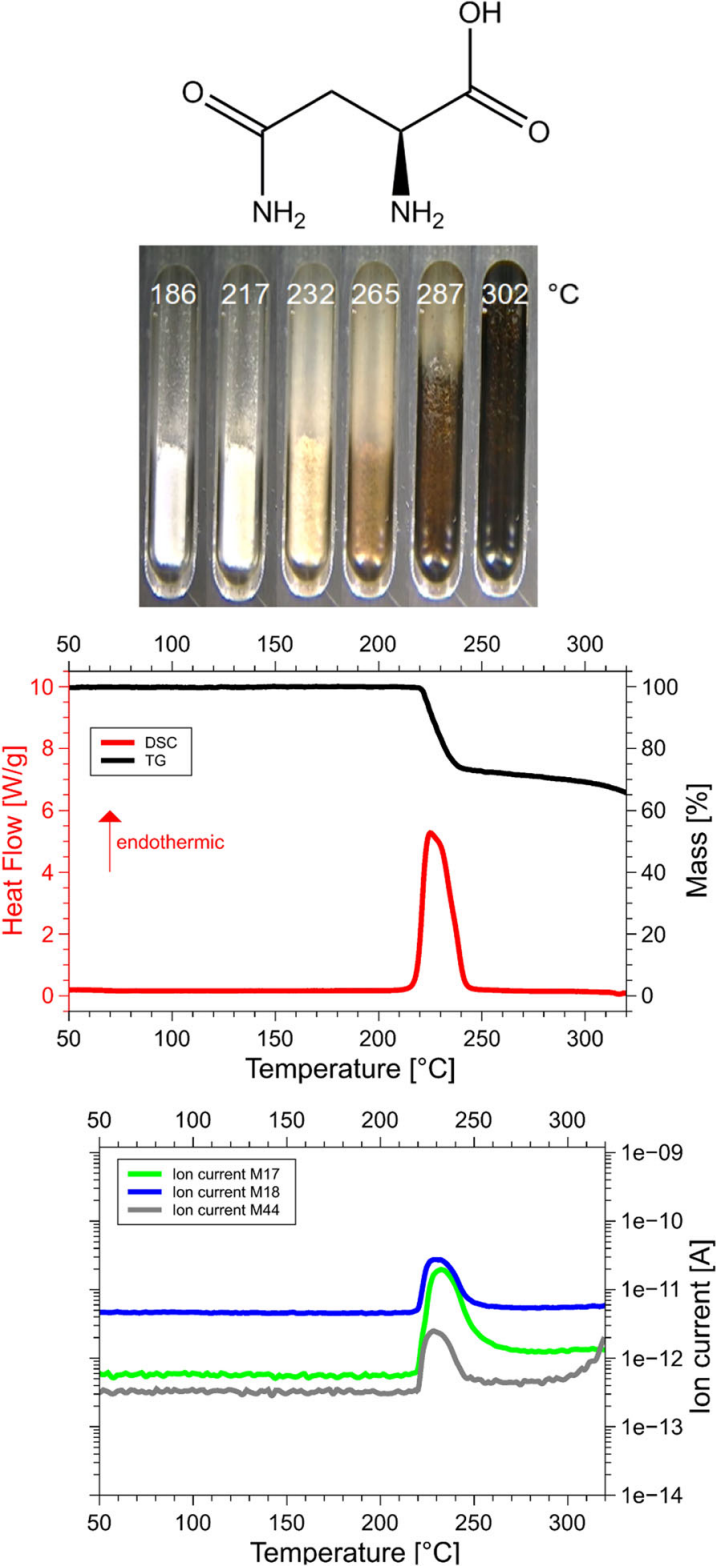
Asparagine data. *C*_4_*H*_8_*N*_2_*O*_3_, 132 Da, *H*_*f*_=−789 kJ/mol

**Fig. 5:**
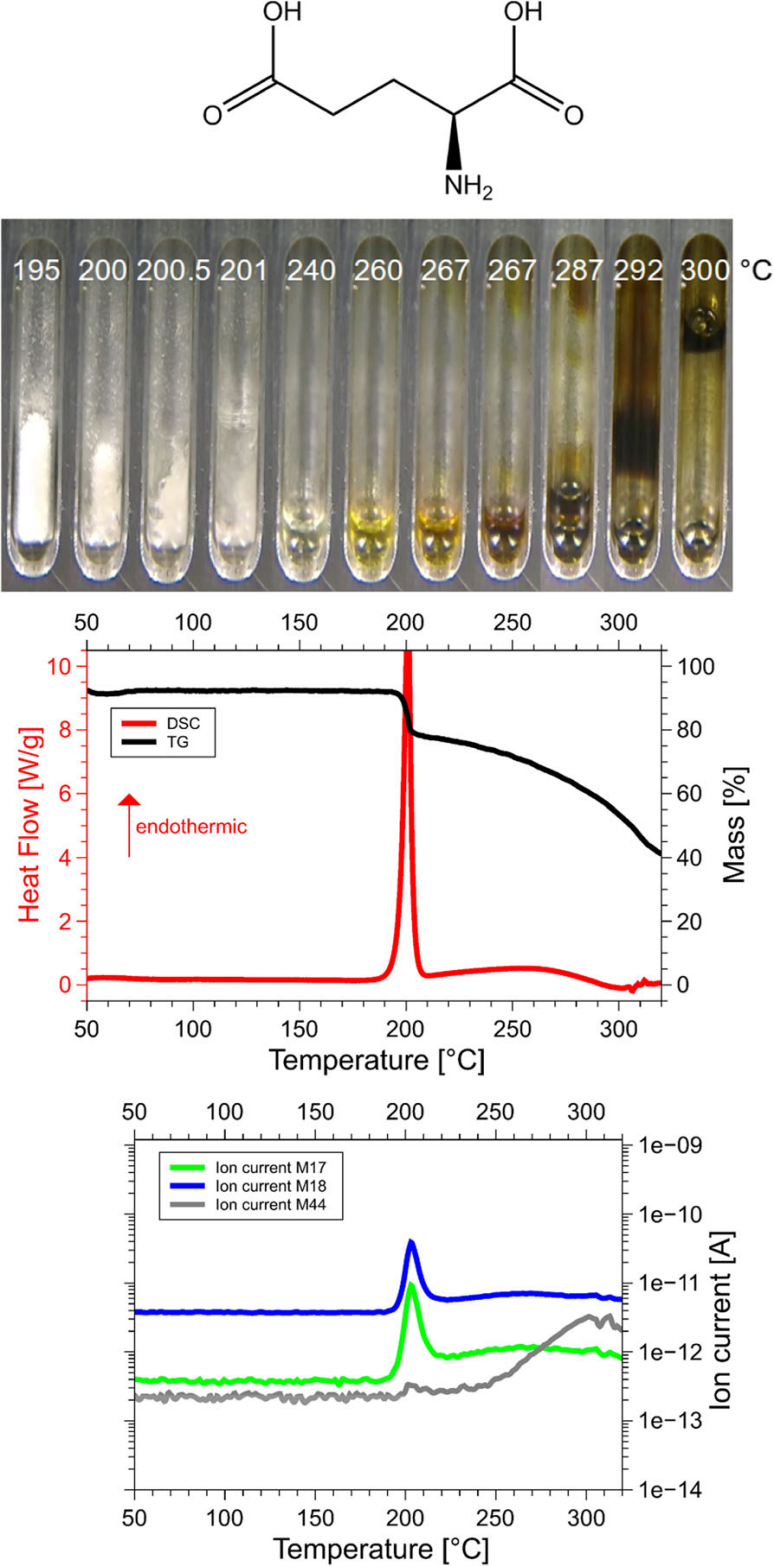
Glutamate data. *C*_5_*H*_9_N*O*_4_, 147 Da, *H*_*f*_=−1097 kJ/mol

**Fig. 6:**
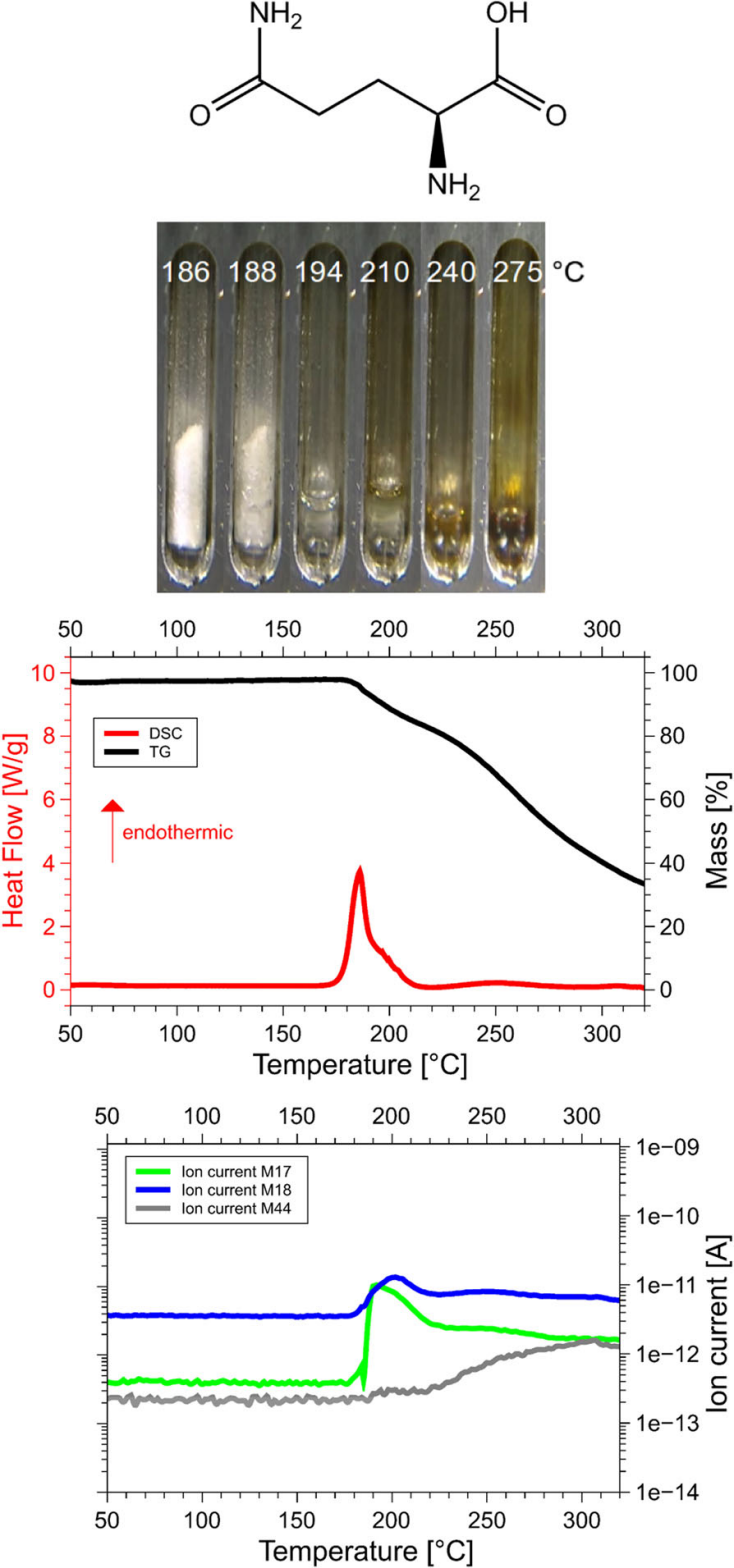
Glutamine data. *C*_5_*H*_10_0*N*_2_*O*_3_, 146 Da, *H*_*f*_=−826 kJ/mol

**Fig. 7:**
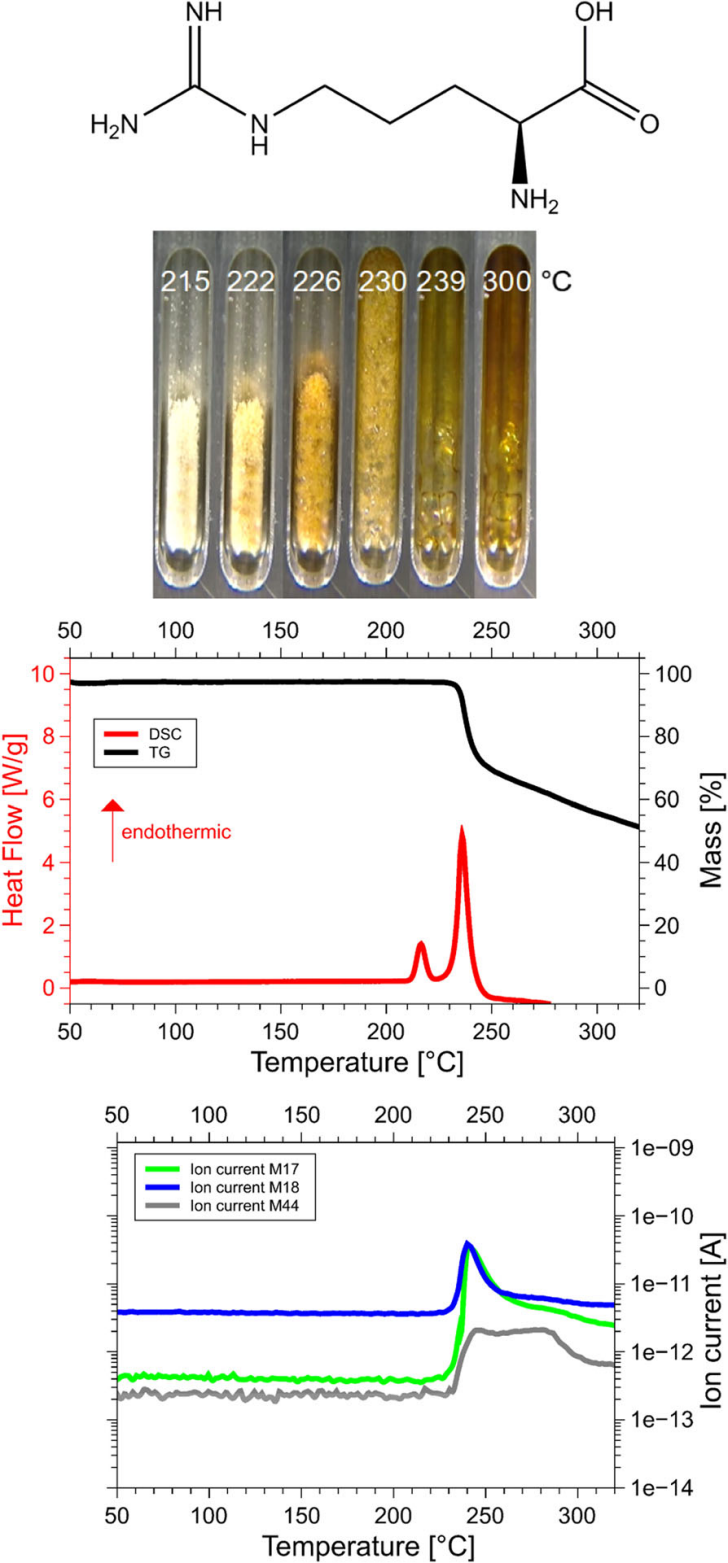
Arginine data. *C*_6_*H*_14_*N*_4_*O*_2_, 174 Da, *H*_*f*_=−623 kJ/mol

**Fig. 8:**
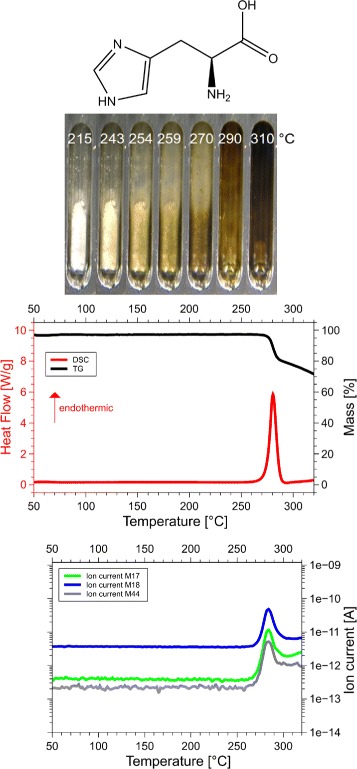
Histidine data. *C*_6_*H*_9_*N*_3_*O*_2_, 155 Da, *H*_*f*_=−467 kJ/mol

Although we examined all 20 amino acids, we report results for those eight of them, for which the sum of the volatile gases, NH_3_, H_2_O, CO_2_ and H_2_S, matched the mass loss registered by thermogravimetry (TG). Only if both, the mass and the enthalpy balance match precisely, as in our case ±5 Da (see Table 1 for details), it is possible to take these data as a proof for the correctness of the proposed reaction. This is the reason why only 8 of the 20 amino acids are reported here. Only for them we know for sure, how they decompose. For each amino acid we show the skeleton structure, the optical observations, the DSC signal in red and the TG signal in black, as well as the ion currents for important channels, quantitatively significant are only the 17 Da (NH_3_, green lines), 18 Da (H_2_O, blue lines), and 44 Da (CO_2_, grey lines) signals. The logarithmic scale overemphasizes the molecular weights. The DSC data are given in W/g, the TG data in %. The QMS data are ion currents [A] per sample. All data are summarized in Fig. 1 for glycine, Fig. 2 for cysteine, Fig. 3 for aspartic adic, Fig. 4 for asparagine, Fig. 5 for glutamic acid, Fig. 6 for glutamine, Fig. 7 for arginine, and Fig. 8 for histidine.

**Table 1:** Data overview

AAA	A	M.W.	H _f_	T _peak_	H _peak_	*Δ*M	QMS-17	QMS-18	QMS-44	M _gas_	*Δ*M - M _gas_	H _res_
		Da	kJ/mol	°C	kJ/mol	Da	mol/mol	mol/mol	mol/mol	Da	Da	kJ/mol
Gly	G	75	−529	250	64	35	0,44 ± 0,01	1,27 ± 0,04	0,07 ± 0,00	34	1	−172
Cys	C	121	−534	245	94	98	0,54 ± 0,05	0,37 ± 0,05	0,99 ± 0,07	59	39	−27
Asp	D	133	−973	256	121	39	0,55 ± 0,04	1,92 ± 0,15	0,03 ± 0,00	45	−6	−473
Asn	N	132	−789	225	118	35	0,97 ± 0,03	1,11 ± 0,05	0,06 ± 0,00	39	−4	−451
Glu	E	147	−1010	201	89	20	0,25 ± 0,02	0,90 ± 0,08	0,00 ± 0,00	21	−1	−779
Gln	Q	146	−826	185	77	22	0,50 ± 0,02	0,53 ± 0,12	0,00 ± 0,00	18	4	−675
Arg	R	174	−624	236	54	47	1,09 ± 0,04	0,87 ± 0,02	0,07 ± 0,01	37	9	−334
His	H	155	−467	280	82	27	0,30 ± 0,01	1,01 ± 0,04	0,10 ± 0,00	28	−1	−169

The three letter codes, and one letter codes, of the amino acids are listed in the first two columns. Their molecular weight is given in units of Dalton [Da] in the third column, the literature values [8] of their heat of formation, H _f_ in [kJ/mol] in the fourth. Columns five and six refer to our experimental calorimetric data: the temperature where the maxima of the peaks occur, T _peak_, in centigrades, and the areas of the endothermic peaks, H _peak_ in [kJ/mol]. The mass spectrometric data follow in columns seven to ten: *Δ*M in Dalton [Da] is the total weight loss in each peak, measured by TGA. QMS-17, given in units [mol/mol], is the mass found in the channel with molecular weight 17 Da, in all likelihood NH_3_. The unit is amount of mols in channel 17-Da per mol amino acid. The column QMS-18 lists the same parameter for the channel with molecular weight 18 Da, in all likelihood H_2_O. The column QMS-44 the same for the channel with molecular weight 44 Da, in all likelihood CO_2_. For example, the value 0.44 in the first line means that 0.44 mol NH_3_ per mol Gly passed through the mass spectrometer. The next column, titled M _gas_, is the calculated sum of the molecular weights found in the 17 Da, plus 18 Da, plus 44 Da channels, i.e. in the first line 34 Da=0.44×17+1.27×18+0.07×44 [Da]. Generally, if there are a, b and c mols of NH_3_, H_2_O and CO_2_, respectively, M _gas_= 17a + 18b + 44c. The penultimate column, *Δ*M-M _gas_, lists the difference of weight loss, *Δ*M measured by DTA and the total mass M _gas_, found as NH_3_, H_2_O and CO_2_. The smallness of *Δ*M-M _gas_ assures that, within error limits, volatile products were only NH_3_, H_2_O and CO_2_. The notable exception is Cystein, which gives off another gas, see “Cysteine” subsections in “Raw data” and “Data analysis, amino acid by amino acid” sections of the text. The last column is the enthalpy of formation of the solid residue, which is the heat of formation H _f_(s) of the solid amino acid [8] minus the heats of formation used to form NH_3_, H_2_O and CO_2_. It is calculated as H _res_ = H _f_−45a −242b −396c

#### Glycine, Gly, G

*C*_2_*H*_5_N*O*_2_, 75 Da, *H*_*f*_= −528 kJ/mol

#### Cysteine, Cys, C

*C*_3_*H*_7_N*O*_2_S, 121 Da, *H*_*f*_ = −534 kJ/mol

#### Aspartic acid, Asp, D

*C*_4_*H*_7_N*O*_4_, 133 Da, *H*_*f*_ = −973 kJ/mol

#### Asparagine, Asn, N

*C*_4_*H*_8_*N*_2_*O*_3_, 132 Da, *H*_*f*_ = −789 kJ/mol

#### Glutamic acid, Glu, E

*C*_5_*H*_9_N*O*_4_, 147 Da, *H*_*f*_ = −1097 kJ/mol

#### Glutamine, Gln, Q

*C*_5_*H*_10_*N*_2_*O*_3_, 146 Da, *H*_*f*_ = −826kJ/mol

#### Arginine, Arg, R

*C*_6_*H*_14_*N*_4_*O*_2_, 174 Da, *H*_*f*_ = −623kJ/mol

#### Histidine, His, H

*C*_6_*H*_9_*N*_3_*O*_2_, 155 Da, *H*_*f*_ = −467 kJ/mol

The DSC, TGA and QMS curves share one essential feature: In DSC there are peaks at a certain temperature T _peak_ for each amino acid, at the same temperatures they are accompanied by drops in TGA and QMS peaks. The simple fact that the DSC and QMS signals coincide in bell shaped peaks with the TGA drop proves that essentially one simple decomposition process takes place, there is not a spectrum of decomposition temperatures, as there would be for proteins. Qualitatively this proves that the process observed is neither melting nor sublimation (as claimed in the literature [5]). The observed process is decomposition, none of the eight amino acids exists in liquid form. The optical observations, not obtained under vacuum but under some air access, are informative nevertheless. Solid/liquid transitions, with the liquid boiling heavily, coincide with the peak temperatures for Gly, Cys, Gln, Glu, Arg and His. Only for Asn and Asp there are solid/solid transformations at the peak temperatures. For Asn there is liquification at 280 °C, Asp stays solid up to 320 °C.

### Calibration and quantitative mass spectrometry

**Fig. 9:**
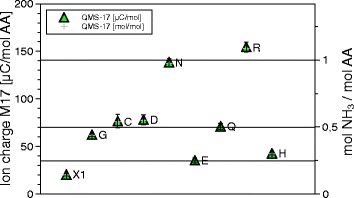
QMS data for the 17 Da channel. Signals in the 17 Da, the NH_3_ channel, for each of the amino acids. Ionic charges in the peaks on the left, mol NH_3_/mol amino acid on the right. The clustering of G, C, D, Q around $\frac {1}{2}$ mol NH_3_ per mol AA and of N and R around 1 mol NH_3_ per mol AA is striking

**Fig. 10:**
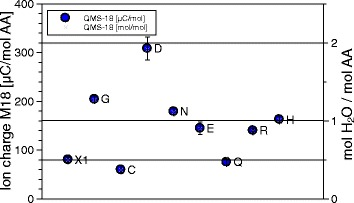
QMS data for the 18 Da channel. Signals in the 18 Da, the H_2_O channel, for each of the amino acids. Ionic charges in the peaks on the left, mol H_2_O/mol amino acid on the right. The clustering of C and Q around the $\frac {1}{2}$ mol H_2_O level, of N, E, R, H around the 1 mol H_2_O level, and the 2 mol point for D are striking

**Fig. 11:**
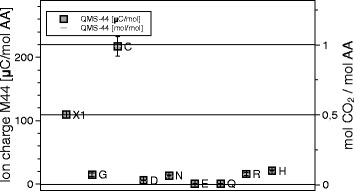
QMS data for the 44 Da channel. Signals in the 44 Da, the CO_2_ channel, for each of the amino acids. Ionic charges in the peaks on the left, mol CO_2_/mol amino acid on the right. Only C produces 1 mol CO_2_, the level of the others is negligible

The DSC signals have the dimension of specific power [W/g], the QMS ones are ion currents of the order of pA. Integration over time, or, equivalently, temperature, gives the peak areas, which are specific energies [J/g] and ionic charges, of the order of pC. Reduction from specimen weights, typically 10 mg, to mol values is trivial. In absolute terms the ion currents and ionic charges are meaningless, because equipment dependent, calibration is needed. Only one reliable calibration substance was available, sodium bicarbonate (NaHCO_3_) = X1. It decomposes upon heating, 2 NaHC*O*_3_ → N*a*_2_C*O*_3_+C*O*_2_+*H*_2_O. The $\mathrm {\frac {1}{2} CO_{2}}$ mol/mol NaHCO_3_ and $\mathrm {\frac {1}{2} H_{2}O}$ mol/mol NaHCO_3_ lines were quantitatively repeatable over months, in terms of pC/mol CO_2_ and pC/mol H_2_O. They served to identify 1 mol CO_2_/mol Cys and $\frac {1}{2}$ mol H_2_O/mol Q beyond any doubt. In the absence of primary NH_3_ calibration we had to resort to secondary substances, glutamine, aspartic acid and asparagine, which retained stable NH_3_ and H_2_O signals over months. The $\frac {1}{2}$ mol NH_3_/mol Q can only come from the glutamine dimer, which implies that also the H_2_O signal from glutamine corresponds to $\frac {1}{2}$ mol H_2_O/mol Q. For the other two, the correspondence between 1 mol H_2_O and 1 mol NH_3_ is convincing. Thus we had four consistent reference points: $\frac {1}{2}$ mol H_2_O and $\frac {1}{2}$ mol CO_2_ from NaHCO_3_, and $\frac {1}{2}$ mol NH_3_ from glu- tamine, and 1 mol NH_3_ from Asparagine. For each amino acid sample, the ion current is measured individually in each mass channel between 1 and 100 Da in 1 Da intervals. Integration over time (and temperature) gives for each mass the ion charge per mol AA, [C/molAA], and with the four calibrations the final values of mol/molAA. In Figs. 9, 10 and 11 the ion charges are plotted on the left, the mol amounts on the right. In the graph for 17 Da (Fig. 9) there appeared a 20 *μ*C/mol signal for the reference substance X1. Since this definitely cannot contain NH_3_, a systematic error of 20 *μ*C/mol must be present, though the statistical errors are smaller.

The absolute ion currents of Figs. 9, 10 and 11 are equipment dependent and not significant, but the relative values are encouraging. One mol NH_3_ produces 12% less and CO_2_ 54% more ions than one mol H_2_O. Indeed the ionization cross sections of NH_3_, H_2_O and CO_2_ are reported to be in that order [6].

**Fig. 12:**
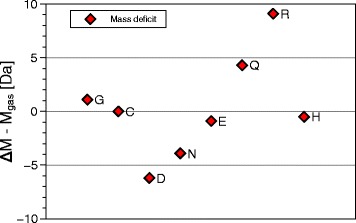
Comparison of mass balances registered by TGA and QMS experiments. The difference between the mass loss registered by TGA, *Δ*M and the volatile mass found as NH_3_, H_2_O, CO_2_ and H_2_S, *Δ*M − M _gas_ remains below |9| Da. This is confirmation that no other gases are produced

Figures 9, 10, 11 and 12 and Table 1 summarize the experimental data: With the exception of cysteine, thermal decomposition results in three gases, mainly H_2_O, less NH_3_ and hardly any CO_2_. The weight of these three gases adds up to the weight loss registered by TGA, therefore no other gases evolve in appreciable amount − they are not seen in QMS either. The proximity of the molfractions to integer or half-integer values indicates simple decomposition chains. The process causing the peaks cannot be melting (because of the mass loss), nor sublimation (because of the QMS signals). One concludes that amino acids do not exist in liquid or gaseous form. They decompose endothermally, with heats of decomposition between −72 and −151 kJ/mol, at well defined temperatures between 185 °C and 280 °C.

### Data analysis, amino acid by amino acid

These amino acids consist of different side chains attached to the C *α* of the same backbone, N*H*_2_−Cα−(*C*^∗^OOH), but their decomposition chains are quite different. The pyrolytic process is controlled by three balance laws: In terms of Da the masses must add up, chemically the atomic species must balance, and the enthalpy of formation must equal the enthalpies of formation of the products plus the endothermic heat of reaction. The amounts of volatile products are experimental values (TGA and QMS). For the residues only the mass is experimental, their composition is inferred. In this section we analyze possible pathways. Although the choices, restricted by compositional, mass and enthalpy considerations, are convincing, they cannot be unique beyond doubt. Alternatives to our proposals, but indistinguishable by us, are possible. Analyses of the decomposition chains are, therefore, tentative or speculative. Nevertheless, they are less speculative than those of Rodante et al. [7], who had only TGA and DSC, but no QMS at their disposal. What Acree and Chickos [5] call “sublimation enthalpies” agree more or less with our decomposition enthalpies. One concludes that they must refer to decomposition, not merely sublimation without composition change.

We made use of the enthalpy values listed for standard conditions [8] without minor corrections for specific heats and entropies up to the actual reaction temperatures. Moreover, hydrogen gas escapes our attention, it is too light (2 Da) to be registered in QMS and TGA, nor does it appear in the enthalpy sum, its heat of formation being zero by definition. With exception of hydrogen, the mass balance, controlled by TGA, confirms that beyond the residue and the three gases, nothing else is formed. The real constraint is the enthalpy balance. For the enthalpy balance production of water is necessary. The expression of the formation enthalpies of the 20 amino acids (C_*a*_H_*b*_N_*c*_O_*d*_S_*e*_) has the least square fit *H*_*f*_(C_*a*_H_*b*_N_*c*_O_*d*_S_*e*_)= 30.3*a* −37.8*b*+16.5*c*−182.4*d*−71.3*e* [kJ/mol]. The oxygens counterbalance the others with −182 kJ/mol. The obvious way of efficiently transferring enthalpy from the reactants to the products is the formation of water, with H_f_ (*H*_2_*O*)=−242 kJ/mol.

Detailed analysis for each amino acid is helped by preliminary reference to a few reactions possible in principle. CO_2_ production in Cys is obviously a special case. In principle one expects the N-termini to be stable, making desamination to produce NH_3_ unlikely. Nitrogen in the side chains is another matter, indeed the NH_3_ producing Asn and Arg have nitrogen in their side chains. The predominance of H_2_O production indicates instability of the C-terminus beyond the C ^∗^ atom, where dehydration can occur by n-oligomerization, which yields (n-1)/n mol H_2_O/mol AS, from dimerization for *n*=2, to 1 mol H_2_O/mol for n →*∞* in polymerization. A special case of dimerization is external cyclization in the diketopiperazine reaction, which yields 1 mol H_2_O/mol AA. These involve joining N- and C- termini in a dehydration reaction. For long side chains also internal cyclization, where the end of the side chain connects to the C-terminus can be envisaged. Integer and half-integer mol values restrict the choice for the residues, but not unequivocally.

All DSC peaks are endothermic, their areas are given negative signs. With this convention endothermic evaporation and exothermic production of water are written as

$$\begin{array}{@{}rcl@{}} {\mathrm{H_{2}O(l)}} &\quad \longrightarrow &\quad {\mathrm{H_{2}O(g)}}\\ \mathbf{-285.5} &&\quad \mathbf{-241.8} \quad \mathbf{-44}~\boldsymbol{kJ/mol} \end{array} $$

$$\begin{array}{ccrcl} {2} (\mathrm{H}_{2})& + & \mathrm{O}_{2} & \longrightarrow & 2 (\mathrm{H}_{2}\mathrm{O})(\mathrm{g})\\ \mathbf{0} & &\mathbf{0}& & 2 \mathbf{(-241.8)} + 2 \mathbf{(241.8)}~\boldsymbol{kJ/mol} \end{array} $$

#### Glycine

Glycine, Gly, G, C_2_H_5_NO_2_, 75 Da, H_f_ =−528 kJ/mol.

Simple endothermic peak at 250 °C, H_peak_ =−72.1 kJ/mol.

**Fig. 13:**
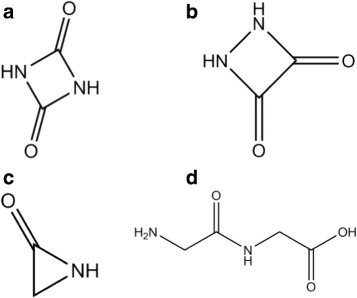
Interpretation of Glycine data. **a**, Residue of Gly, *C*_2_*H*_2_*N*_2_*O*_2_, 1,3-Diazetine-2,4,dione, 86 Da. **b**, Isomer of Fig. 13a, 1,2-Diazetine-3,4-dione, 86 Da. **c**, 2-Aziridinone, *C*_2_*H*_3_NO, 57 Da. **d**, Intermediate dimer, glygly, *C*_4_*H*_8_*N*_2_*O*_3_, 132 Da

The QMS signal of $\frac {3}{2}$ mol H_2_O/mol Gly plus $\frac {1}{2}$ mol NH_3_/mol Gly is beyond doubt, it is confirmed by the mass loss of 35 Da /mol Gly. This leaves only 10% of the original hydrogen for the residue. The triple and double bonds in carbon rich C_4_HNO, C_3_HNO and C_2_HNO preclude them enthalpy wise and make deposition of carbon likely,

$$\begin{array}{lclcccrcccl} {4 (\mathrm{C}_{2}\mathrm{H}_{5}\text{NO}_{2})} & \longrightarrow & {6\,(\mathrm{H}_{2}\mathrm{O})} & + & 2 {(\text{NH}_{3})} & + & {6\,\mathrm{C}} & + & 2 &{ (\text{CHNO})} \\ \mathbf{-2112} & & \mathbf{-1452}&&\mathbf{-92}& &\mathbf{0} & & 2 & \times & \mathbf{-288}~\boldsymbol{kJ/mol}, \end{array} $$

leaving -280 kJ/mol = x for the moiety CHNO, which is the composition of the peptide bond. The database ChemSpider [9] lists two symmetric molecules consisting entirely of peptide bonds, 1,3-Diazetine-2,4-dione, C_2_H_2_N_2_O_2_, chemspider 11593418, 86 Da, (Fig. 13a) or its isomer 1,2-Diazetine-3,4-dione, C_2_H_2_N_2_O_2_, chemspider 11383421, 86 Da, Fig. 13b. The scarcity of hydrogen is such that not even the smallest lactam, 2-Aziridinone, C_2_H_3_NO, 57 Da, chemspider 10574050, bp 57 °C (Fig. 13c) can serve as residue.

$$\begin{array}{lclclclclll} 2 \text{Cys} = \\ \mathrm{C}_{6}\mathrm{H}_{14}\mathrm{N}_{2}\mathrm{O}_{4}\mathrm{S}_{2} & \longrightarrow & 2\,\text{CO}_{2} & + & 2\, \mathrm{H}_{2}\mathrm{S} & + & \text{NH}_{3} & + & \mathrm{C}_{4}\mathrm{H}_{7}\mathrm{N}, &\\ \ \ \ 2\mathbf{(-534)} & &\! \mathbf{-788} & & \mathbf{-40} & & \mathbf{-46} & & \mathbf{-47} &\!\!\! + & 2\,\mathbf{(-96)}~\boldsymbol{kJ/mol}. \end{array} $$

The simplest pathway seems the formation of linear Glycylglycine, chemspider 10690 (Fig. 13d), m.p. 255 °C (which is T _peak_!) C_4_H_8_N_2_O_3_, *H*_*f*_(s) = − 748 kJ/mol [8] from which the central peptide bond −(C =O)-NH − is detached by cutting off the NH_2_-C *α*-H_2_ − group on one, and the C *α*-H_2_-C ^∗^OOH group on the other side. The former makes NH_3_ plus C, the latter makes 2 C plus 2 H_2_O. This process is specific for the glycine dimer, in which the C ^*α*^ atoms are not protected by proper sidechains, they are just −*Cα*-H_2_ − units. This pathway to shear peptide bonds is of interest in the context of possible peptide nucleic acid (PNA) synthesis [10] via N-2-aminoethylglycine (AEG), C_4_H_10_N_2_O_2_, chemspider 379422, which is deoxidized diglycine, 2Gly→*O*_2_+AEG.

#### Cysteine

Cysteine, Cys, C, C_3_H_7_NO_2_S: 121 Da, H_f_ =−534 kJ/mol.

T_peak_ = 221 °C with a mass loss of 98 Da, H_peak_=−96 kJ/mol.

The clear 1 mol CO_2_ signal leaves no oxygen to form H_2_O, therefore the spurious 18 Da line must stem from a systematic error. There is also $\frac {1}{2}$ mol NH_3_/mol Cys. For H_2_S there is indeed a signal at 34 Da. It corresponds to 1 mol, because the ionization cross sections of H_2_S and H_2_O are nearly identical, so that the calibration of Fig. 10 applies. The mass loss of 44+34+8.5=70% of 121 Da agrees with TGA. Chemical analysis found no sulfur in the residue. No possibility for forming disulfide bridges between molecules is left. Neither COS nor CS_2_ was found. The total reaction is

**Fig. 14:**
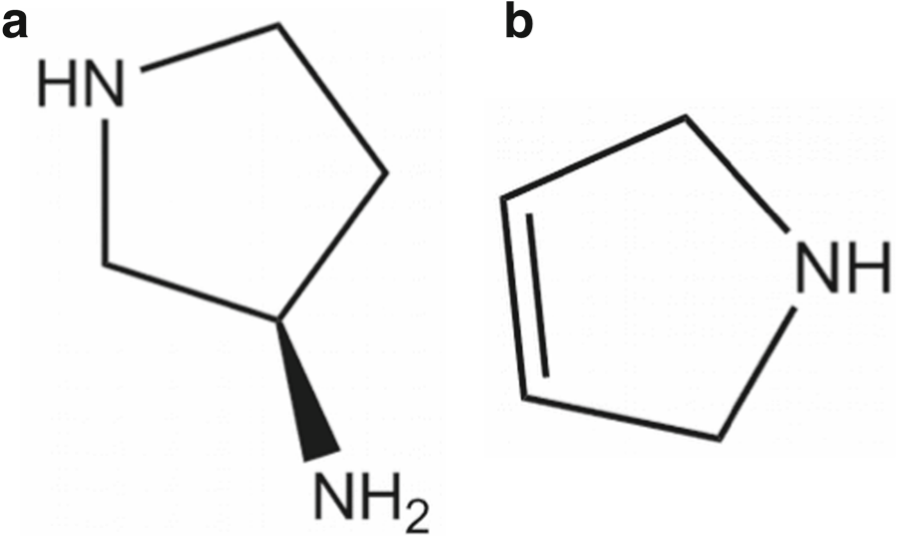
Interpretation of Cysteine data. **a**, Intermediate compound: 3-pyrrolidinamine, chemspider 144134, 86 Da. **b**, Residue of Cys, *C*_4_*H*_7_N, 2,5-Dihydro-1H-pyrrole, chemspider 13870958, 69 Da

On the left −1068 kJ, on the right −1113 kJ. Pathway to the formation of C_4_H_7_N might be ejection of the carboxyl group −*C*^∗^OOH and the −SH group from Cys, the remaining chain NH_2_-C *α*-C ^∗^ is too short for internal, but suitable for external cyclization. Two of these form the asymmetric 5-ring (3-pyrrolidinamine, chemspider 144134), Fig. 14a, from which the −NH_2_ is cutt off. Indeed the $\frac {1}{2}$ NH_3_ ejected confirms such dimerization. That leaves the molecule C_4_H_7_N: 2,5-Dihydro-1H-pyrrole, chemspider 13870958, b.p. 90 °C, 69 Da, *H*_*f*_(s) =−46.6 kJ/mol (Fig. 14b), or another pyrroline, with the double bond elsewhere in the ring. Indeed there is heavy boiling beyond the peak. In view of the richness in hydrogen, several small hydrocarbon lines are not surprising.

#### Aspartic acid

Aspartic acid, Asp, D, C_4_H_7_NO_4_:133 Da, H_f_ =−973 kJ/mol.

DSC shows two distinct peaks, at 230 °C and at 250 °C, in each of which 1 mol H_2_O/mol Asp is ejected. The endothermic heats are -64 and -61 kJ/mol, respectively. The substance stays a powder up to 294 °C, i.e. solid/solid transformation in the peak. The reaction

$$\begin{array}{llllllcl} \mathrm{C}_{4}\mathrm{H}_{7}\text{NO}_{4} & \longrightarrow & \mathrm{H}_{2}\mathrm{O} & + & \mathrm{H}_{2}\mathrm{O} & + & \mathrm{C}_{4}\mathrm{H}_{3}\text{NO}_{2}\\ \mathbf{-973} & & \mathbf{-242} & &\mathbf{-242} & & y &\!\! \mathbf{(-125)}~\boldsymbol{kJ/mol}, \end{array} $$

with calculated *y*=−364 kJ/mol, which is reasonable for the formation enthalpy of the polysuccinimide unit (PSI). The molecular weight of C_4_H_3_NO_2_ is 97 Da.

**Fig. 15:**
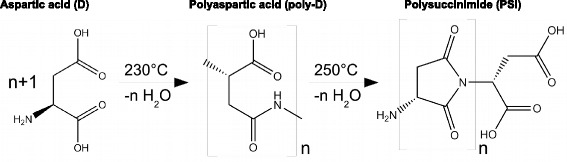
Interpretation of Aspartate data. The pathway from Aspartic acid (D) to polysuccimide (PSI). Compared with succinimide, the N-C bond in polysuccinimide economizes two hydrogen atoms

The compound (C_4_H_3_NO_2_) _*n*_ is polysuccinimide. The two peaks prove that the reaction occurs in two steps, in the first at 230 °C the condensation reaction produces polyaspartic acid, n Asp→H_2_O+(*Asp*)_*n*_, in the second at 250 °C the poly-Asp degrades to polysuccinimide (PSI) by ejection of another 1 mol H_2_O/mol Asp. Such a reaction was reported by Schiff [11]. The molecule drawn in Fig. 15 is *β*-poly-Asp, there is an isomer, *α*-poly-Asp, where the next C in the ring forms a bond to its neighbour. We have no possibility to decide between the two.

#### Asparagine

Asparagine, Asn, N, C_4_H_8_N_2_O_3_: 132 Da, H_f_ =−789 kJ/mol.

**Fig. 16:**
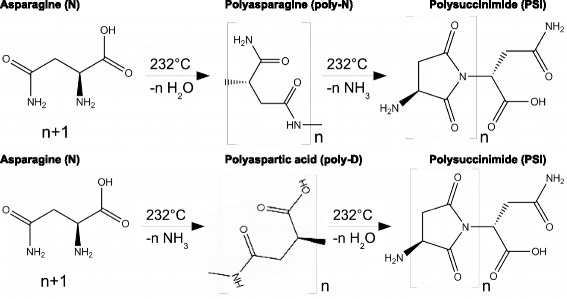
Interpretation of Asparagine data. Two pathways from asparagine (N) to polysuccinimide (PSI): either through polyasparagine (poly-N) or polyaspartic acid (poly-D). Compared with succiminide, the N-C bond in polysuccinimide economizes two hydrogen atoms

In the broad peak at 232 °C, 1 mol H_2_O /mol Asn and 1 mol NH_3_ /mol Asn are ejected. H_peak_=−122 kJ/mol. The product stays a white powder up to 265 °C, i.e. there is a solid/solid transformation in the reaction

$$\begin{array}{llllllcl} \mathrm{C}_{4}\mathrm{H}_{8}\mathrm{N}_{2}\mathrm{O}_{3} & \longrightarrow & \mathrm{H}_{2}\mathrm{O} & + & \text{NH}_{3} & + & \mathrm{C}_{4}\mathrm{H}_{3}\text{NO}_{2} & \\ \mathbf{-789} & & \mathbf{-242} & & \mathbf{-46} & & \times &\!\! \mathbf{(-122)}~\boldsymbol{kJ/mol}, \end{array} $$

with calculated *x*=−379 kJ/mol. In the Asp decomposition, *H*_*f*_(C_4_H_3_NO_2_) was calculated as *y*=−364 kJ/mol. The two values agree, although because of their histories, the two PSI are not identical. If Asn followed the example of Asp, it would eject 1 mol H_2_O /mol Asn in the condensation reaction n Asn→*H*_2_O+(*Asn*)_*n*_, poly-N, followed by degradation of poly-N to polysuccinimide (PSI) by ejection of 1 mol NH_3_ /mol Asn. If, however, the H_2_O of the condensation reaction is not ejected but retained, it can replace the −NH_2_ in poly-N by −OH. According to Asn−>NH_3_+ poly-D, this amounts to the formation of polyaspartic acid from asparagine by ejection of NH_3_. The poly-D then degrades to polysuccinimide (PSI) by ejection of 1 mol H_2_O/mol Asn. Apparently both alternatives shown in Fig. 16 occur, and there is one broad peak containing both NH_3_ and H_2_O.

Though the formulae for PSI formed from Asp and from Asn, are the same, (C_4_H_3_NO_2_) _*n*_, these two residues need not be identical. For kinetic reasons the oligomerization or polymerization might have proceeded to different lengths in poly-Asp and poly-Asn, therefore the degraded products PSI might have different lengths, with different stabilities and melting points. Moreover, the telomers are different, −OH for PSI from Asp and −NH_2_ for PSI from Asn. Indeed PSI from Asp remains a white powder up to 289 °C, while PSI from Asn starts melting at 289 °C.

#### Glutamic acid

Glutamic acid, Glu, E, C_5_H_9_NO_4_:147 Da, *H*_*f*_=−1097 kJ/mol; T_peak_=200°C, H_peak_=−88 kJ/mol.

**Fig. 17:**
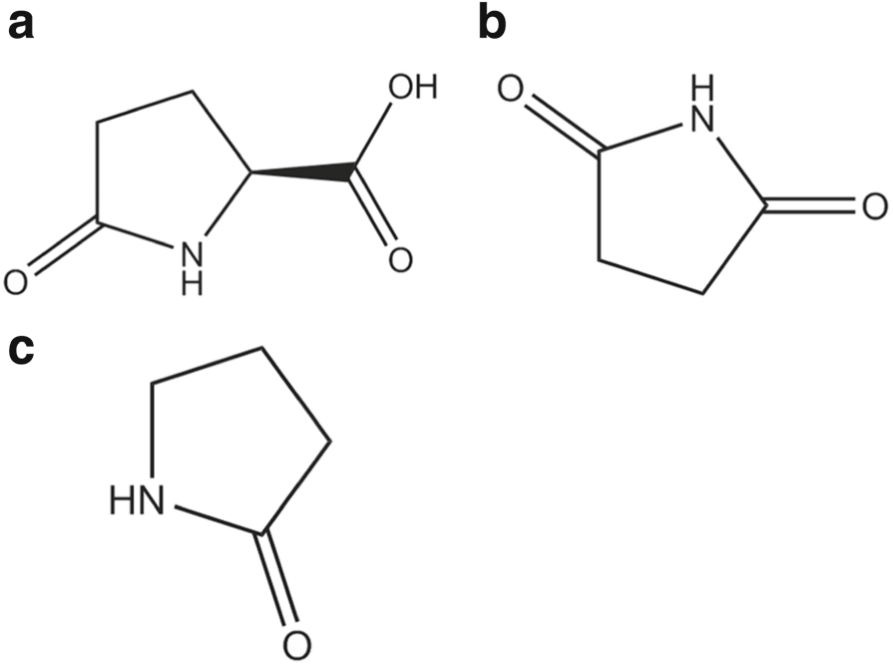
Interpretation of Glutamate data. **a**, The final residue of Glu, pyroglutamic acid, *C*_5_*H*_7_N*O*_3_, 129 Da. **b**, Succinimide, *C*_4_*H*_5_N*O*_2_, 99 Da. **c**, Pyrrolidone, *C*_4_*H*_7_NO, 85 Da

At 200 °C, 1 mol H_2_O /mol Glu is seen in QMS, the DSC area is −121 kJ/mol, mass loss in the peak is 12% (17 Da). The dehydration of Glu has been known for a long time [12].

$$\begin{array}{llllcl} \text{C5H9NO4} & \longrightarrow & \text{H2O} & + & \text{C5H7NO3(l)} &\\ \mathbf{-1097}& & \mathbf{-242}& & \times& \mathbf{(-121)}~\boldsymbol{kJ/mol}, \end{array} $$

with calculated *x*=−734 kJ/mol for H_f_(C_5_H_7_NO_3_), pyroglutamic acid, chemspider 485, 129 Da, T _m_=184 °C, b.p. = 433 °C (Fig. 17a). This lactam is biologically important, but its enthalpy of formation is apparently not known. Known is the *H*_*f*_(s) =−459 kJ/mol and *H*_*f*_(g) =−375 kJ/mol for C_4_H_5_NO_2_, succinimide, 99 Da, the five ring with O= and =O as wings (the structure is like pyroglutamic acid, but with the carboxyl group −COOH replaced by =O, shown in Fig. 17b). The additional O should add about −200 kJ/mol, which makes the −734 kJ/mol for pyroglutamic acid plausible. The TGA weight loss beyond the peak is evaporation. Pyroglutamic acid is formed by inner cyclization of E: after the −OH hanging on C ^*δ*^ is ejected, the C ^*δ*^ joins the −NH_2_ hanging on C ^*α*^. This was suggested by Mosqueira et al. [13], ours is the first experimental evidence for this process. Since QMS does not show any CO_2_, the reaction to C_4_H_7_NO, the lactam pyrrolidone (Fig. 17c), 85 Da, T_m_=25 °C, b.p. = 245 °C, *H*_*f*_(l) =−286 kJ/mol, yellow liquid, can be ruled out, although cutting off the CO_2_ is sterically tempting.

#### Glutamine

Glutamine, Gln, Q, C_5_H_10_N_2_O_3_: 146 Da, H_f_ =−826 kJ/mol.

**Fig. 18:**
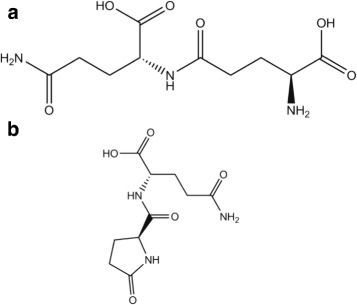
Interpretation of Glutamine data. **a**, Intermediate step, gamma-glutamylglutamine, *C*_10_*H*_17_*N*_3_*O*_6_, 275 Da. **b**, The residue of Gln: 5-Oxo-L-prolyl-L-glutamine, *C*_1_0*H*_1_5*N*_3_*O*_5_, 257 Da

The precise $\frac {1}{2}$ mol fractions of H_2_O and NH_3_ in the peak at T_peak_=185 °C, H_peak_=−77 kJ/mol, indicate that a dimer serves as intermediate step, *γ*-glutamylglutamine (Fig. 18a), C_10_H_17_N_3_O_6_, chemspider 133013, b.p. 596 °C:

$$\mathrm{2\ Q\ = {C_{10}H_{20}N_{4}O_{6}\ \longrightarrow [NH_{3} + C_{10}H_{17}N_{3}O_{6}]}.} $$

After further ejection of H_2_O the total reaction is

$$\mathrm{2\ Q\ = 2 ({C_{5}H_{10}N_{2}O_{3})\ \longrightarrow NH_{3} + H_{2}O + C_{10}H_{15}N_{3}O_{5}.}} $$

The database ChemSpider [9] lists for the residue a suitable molecule, 9185807, 5-Oxo-L-prolyl-L-glutamine (Fig. 18b), *C*_10_*H*_15_*N*_3_*O*_5_, 257 Da, b.p. 817 °C, H_vap_=129 kJ/mol. Above the peak at 185 °C optical observations show indeed a nonboiling liquid, agreeing with the high boiling point quoted.

#### Arginine

Arginine, Arg, R, C_6_H_14_N_4_O_2_: 174 Da, H_f_ =−623 kJ/mol.

**Fig. 19:**
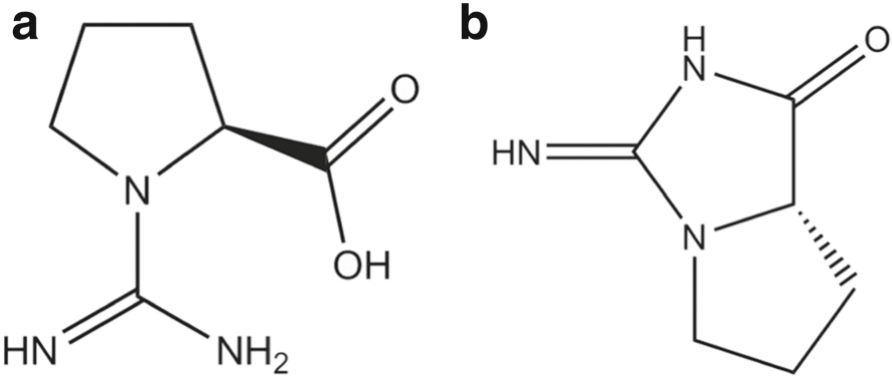
Interpretation of Arginine data. **a**, 1-Carbamimidoylproline, 157 Da, representing the intermediate step after ejection of N*H*_3_ from Arg. **b**, The final residue of Arg, *C*_6_*H*_9_*N*_3_O, 139 Da, “creatine-proline”. The creatine ring on top joins the proline ring

A small peak without mass loss at 220 °C, −14 kJ/mol, and a main peak at 230 °C, −52 kJ/mol, producing 1 mol NH_3_ plus 1 mol H_2_O in QMS, confirmed by the weight loss of 20% of 174 Da in TGA. The precursor peak without mass loss at 220 °C, −14 kJ/mol, probably comes from a rearrangement in the guanidine star. In the large peak a double internal cyclization occurs. The loss of the amino group −NH_2_ in the backbone, and internal cycling joining the N next to the C ^*δ*^ in the side chain to C ^*α*^,

$$\mathrm{C_{6}H_{14}N_{4}O_{2}\ \longrightarrow NH_{3} + H_{2}O + C_{6}H_{11}N_{3}O_{2}.} $$

forms an intermediate, 1-Carbamimidoylproline, 157 Da, chemspider 478133 (Fig. 19a). It is called “..proline”, because the ring is spanned between an N and C ^*α*^, though the N is not from the backbone. By losing the −OH and a second inner cyclization joining the =NH or the −NH_2_ to C ^∗^, one or the other tautomer of the final residue is formed. The total reaction is *C*_6_*H*_14_*N*_4_*O*_2_ →N*H*_3_+*H*_2_O+*C*_6_*H*_9_*N*_3_O, drawn in Fig. 19b, not quoted in the database [9, 14].

This final residue is remarkable. It contains the proline ring, the guanidine star and a peptide bond in the ring of creatinine, which is the 5-ring with the =O and =OH double bonds. Creatinine, H_f_=−240 kJ/mol, m.p. 300 °C, *C*_4_*H*_7_*N*_3_O, chemspider 568, has several tautomeric forms. The end product in question might contain either of those rings. We have no way to decide between the alternatives, but a double ring structure seems likely.

#### Histidine

Histidine, His, H, *C*_6_*H*_9_*N*_3_*O*_2_: 155 Da, *H*_*f*_=−466 kJ/mol.

The QMS results are clear, His ejects 1 mol H_2_O in the reaction

$$\mathrm{His = C_{6}H_{9}N_{3}O_{2} \ \longrightarrow 1\,H_{2}O + C_{6}H_{7}N_{3}O}. $$

**Fig. 20:**
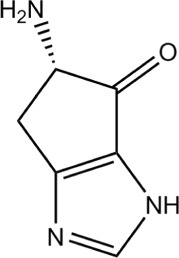
Interpretation of Histidine data. Final residue of His, *C*_6_*H*_9_*N*_3_O, 139 Da, consisting of two 5-rings: 2-amino-2,4-cyclopentadien-1-one (*C*_5_*H*_5_NO, chemspider 28719770) and imidazole

The observed 1mol H_2_O /mol His, confirmed by the weight loss of 13% of 155 Da, could stem from the condensation reaction of polymerization, but the volatility seen optically contradicts this option. Inner cyclization seems likely. If the C ^∗^ of the backbone joins the C of the imidazole ring, with =O and −NH_2_ attached outside, the 5-ring formed joins the 5-ring of the imidazole. The proposed structure is shown in Fig. 20.

The MolPort database [14] quotes this structure, but with the pyrazole ring (where the two N are nearest neighbours) instead of the imidazole ring (where the two N are next nearest neighbours): 5-amino-4H,5H,6H-pyrrolo[1,2-b]pyrazol-4-one, molport 022-469-240. Parting the nitrogens is energetically favorable: for pyrazole *H*_*f*_(s) = +105 kJ/mol, *H*_*f*_(g) = +179 kJ/mol; for imidazole *H*_*f*_(s) = +49 kJ/mol, *H*_*f*_(g) = +132.9 kJ/mol [8]. Moreover, the original His has an imidazole and not a pyrazole ring, and so does the residue.

## Discussion

### Entropy of decomposition

In the tables of Domalski [15], Chickos and Acree [16] and Acree and Chickos [17], at temperatures coinciding with our peak temperatures, “Heats of sublimation” of the order of our endothermic peak areas are reported. Our QMS signals prove that chemical decomposition is involved, but that should have been obvious from the DSC data alone. The average of the entropies of transformation, *S*_peak_=*H*_peak_/*T*_peak_, is 215 J/Kmol, way above the usual entropies of melting (22 J/kmol for H_2_O, 28 for NaCl, 36 for *C*_6_*H*_6_), and higher than typical entropies of evaporation (41 J/Kmol for H_2_O, 29 for C*S*_2_, 23 for C*O*_2_, 21 for N*H*_3_). The endothermic heats in the peaks are therefore neither enthalpies of fusion nor enthalpies of sublimation, they are heats of reaction accompanied by phase changes. There is transformation and decomposition, but no reversible melting. Amino acids are stable in solid form, but not as liquids or gases.

### Peptide bond formation

**Fig. 21:**
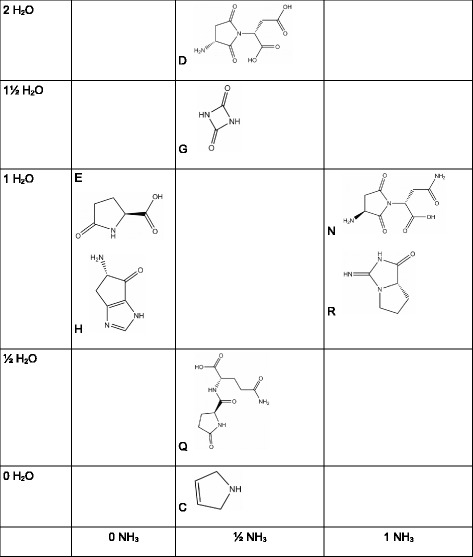
Overview of the residues with respect to H_2_O or NH_3_ contents. All residues are obtained by ejection of 0, $\frac {1}{2}$, 1, $1\frac {1}{2}$ or 2 mols of H_2_O or NH_3_, placing them on two axes. All residues contain either 0, $\frac {1}{2}$ or 1 mol NH_3_ or H_2_O, placing them on two axes. Most of them contain peptide bonds. The polysuccinimide of D and N is an exception, cysteine, for lack of oxygen, the other

Five of the eight amino acids have residues containing peptide bonds, −C(=O) −NH−, only Asp and Asn leave polysuccinimide (PSI), Cys leaves cyclic pyrrolines. The preponderance of water in thermal decomposition is not surprising. In natural protein formation, each participating amino acid suffers damage. In the condensation reaction, where the N-terminus of one molecule reacts with the C-terminus of its neighbour, the planar peptide bond −Cα − CO − N − Cα− is formed. The N-atoms on, and the keto-bound O −atoms off the backbones retain their position. H_2_O is ejected, but neither NH_3_ nor CO_2_ are produced in protein formation. Thermal decomposition of amino acids is analogous. In protein formation, the endothermic heat is provided by ATP, in amino acid decomposition it is thermal energy. Figure 21 summarizes the results.

### Peak areas

Quantitatively, the parallel between protein formation and pyrolysis is confirmed on the enthalpy level. In the formation of a dipeptide, X+Y →*H*_2_O+(X−Y), the difference between the enthalpies of the reactants and the products go into the formation of the peptide bond (PB): *H*_*f*_(X)+*H*_*f*_(Y)= −242 kJ+*H*_*f*_(X−Y)+*H*_*PB*_. With the tabulated value [8, 15] for *H*_*f*_(X), *H*_*f*_(Y) and *H*_*f*_(X-Y) one calculates H _PB_=−67 kJ in glycylglycine, −70 kJ in alanylglycine, −43 kJ in serylserin, −78 kJ in glycylvaline, −65 kJ in leucylglycine, −91/2 kJ in triglycylglycine, −86/2 kJ in leucylglycylglycine, and −58 kJ in glycylphenylalanine. The average value is −59 ± 13 kJ per peptide bond. The narrow standard deviation indicates that the enthalpy of forming a peptide bond is insensitive to its environment, therefore the endothermic values of oligomerization or polymerization should be close to this. One concludes that the formation of a peptide bond in a linear dimer is endothermic with an enthalpy of 59 ± 13 kJ. It is tempting to compare this with the areas of the DSC peak, the observed endothermic heat of the decomposition reaction. The average of the eight amino acids is −105 ± 27 kJ/mol. One concludes that essentially the endothermic heat of decomposition, the peak area, goes into peptide bond formation.

### Production of NH_3_

In cases where the N-terminus, untouched by the condensation, remains attached to a cyclic product, it could be cut off as NH_3_, contributing up to $\frac {1}{2}$ mol NH_3_/mol AA. Remarkable is the absence of methane (C*H*_4_, 16 Da), hydrogen cyanide (HCN, 27 Da) and formamide (C*H*_3_NO, 45 Da), all in mass channels where we would have seen them. These, suspected in prebiotic synthesis of amino acids, do not appear in their decomposition. Although at most only three molecules are involved, two gases and one monomolecular residue, identification of the structure of the latter is not unequivocal, there remain more or less probable other possibilities than our choices. Clearly, without QMS, data from DSC and TGA could not possibly suffice to identify decomposition chains.

### Water, cyclic compounds and peptide bonds

The novel quantitative results emphasize the importance of water and cyclic condensates containing peptide bonds. All postulated residues are cyclic compounds, five of the 8 contain peptide bonds. The residues are stable at temperatures >180 °C and beyond the respective peak termperatures. These facts put constraints on hypothetical origin, state and stability of amino acids in the range between 200 °C and 300 °C in the absence and presence of water, but literature is sparse in that respect. The history of diketopiperazine and derivatives is extensively reviewed by Prasad [18] back until 1888, the synthesis of cyclo-Gly-Gly by Curtius and Gloebel [19], but Prasad emphasized the relevance of these compounds as a class of natural products no earlier than 1922 [20–22]. Today, CDPs are recognized as “transkingdom signaling molecules” [23], indicating highly conserved mechanisms from earliest stages of life on earth. The potential of CDPs and related substances as novel drugs for biomedical applicatons is comprehensively reviewed by Borthwick [24], though he does not cover aqueous regimes above 150 °C. Thermal formation of cyclo-Leu-Leu from the Leu-Leu dipeptide in the solid state was reported to occur at 177 °C [25] − actually several month after the release of QMS data for amino acids Gly, Cys, Asp, Asn, Glu, Gln, Arg, and His in the solid state [26]. There might be differences in terms of thermal cyclization by dehydration, depending on whether or not the origin is an amino acid crystal or a dipeptide. Controlled biosynthesis of CDP by highly conserved enzymes is found in all domains of life [27]. However, the biochemistry of cyclo-dipeptides and related enzymatic pathways is a comparatively unexplored interdisciplinary field, usually based on genome analyses. Just recently, their presence in extremophilic organisms has been highlighted in more detail [28, 29]. Previously reported evidences along with the first conclusive demonstration of thermal cyclization of Gly, Cys, Asp, Asn, Glu, Gln, Arg, and His by QMS, DSC and TGA as reported here put emphasis on the fact that cyclic dipeptides or cyclic compounds could represent thermally more stable precursors of prebiotic life.

## Conclusions

Our comparative analysis allowed us to identify the eight of the twenty standard amino acids, for which the thermochemical equations unequivocally agree with stoichiometric release of NH_3_, H_2_O and CO_2_. The predominance of the release of H_2_O during the process of decomposition instead of melting indicates a common principle of condensation and, depending on the individual properties of the respective intermediate products, subsequent decomposition of the condensation products. Comparative data for all 20 standard amino acids, obtained by complementing DSC and TGA with quantitative mass spectrometry (QMS), have never been reported. For the eight with data closure we can say: Amino acids decompose thermally, they do not sublimate, nor do they melt. Only three gases are formed, mostly H_2_O, less so NH_3_ and hardly any CO_2_. Cys forms *H*_2_S, but not C*S*_2_. In all amino acids investigated, Gly, Cys, Asn, Asp, Gln, Glu, Arg, His, the liquid or solid residues are lactams and heterocyclic compounds with 5- or 6-membered non- (or only partially) aromatic rings, containing one or two nitrogen atoms (pyrrolidines, piperidines, pyrrazolidines, piperazines), most of them with peptide bonds present.

In summary, this work addresses an important question of amino acid thermal stability. Several processes may occur upon heating, chemical decomposition or sublimation/evaporation without decomposition. The aim of this work was to accurately determine these processes. For 8 out of the 20 standard amino acid, we demonstrated that these have a well defined temperature of decomposition. The contemporary detection of products of 17 Da, 18 Da, and 44 Da in the gas phase is the proof for decomposition, concise mass and enthalpy balances do not leave any room for speculation. Analysis and interpretation rule out the existence of any byproduct hydrocarbons which could not have been validated. The analysis for glycine, cysteine, aspartic acid, asparagine, glutamic acid, glutamine, arginine and histidine is beyond any doubt. At a heating rate of 5 K/min, neither melting nor sublimation take place. At least 8 of 20 standard amino acids do not exist in liquid form.
